# Ancestry-dependent genetic structure of the Xq28 risk haplotype in the Mexican population and its association with childhood-onset systemic lupus erythematosus

**DOI:** 10.3389/fmed.2022.1044856

**Published:** 2023-01-12

**Authors:** Humberto García-Ortiz, Francisco Barajas-Olmos, Marlen Flores-Huacuja, Monserrat I. Morales-Rivera, Angélica Martínez-Hernández, Vicente Baca, Cecilia Contreras-Cubas, Lorena Orozco

**Affiliations:** ^1^Immunogenomics and Metabolic Diseases Laboratory, National Institute of Genomic Medicine, SS, Mexico City, Mexico; ^2^Department of Rheumatology, Hospital de Pediatría, CMN Siglo XXI IMSS, Mexico City, Mexico

**Keywords:** childhood-onset systemic lupus erythematosus, Xq28 risk haplotype, ancestry-dependent, *IRAK1*, *MECP2*

## Abstract

**Objective:**

Here we aimed to investigate the association of the Xq28 risk haplotype (H1) with susceptibility to childhood-onset systemic lupus erythematosus (SLE), and to compare its frequency and genetic structure in the Mexican population with those in other continental populations.

**Methods:**

We genotyped 15 single-nucleotide variants (SNVs) that form the H1 haplotype, using TaqMan real-time PCR. The association analysis [case-control and transmission disequilibrium test (TDT)] included 376 cases and 400 adult controls, all of whom were mestizos (MEZ). To identify risk alleles in Mexican Indigenous individuals, SNVs were imputed from whole-exome sequencing data of 1,074 individuals. The allelic frequencies determined in MEZ and Indigenous individuals were compared with those of the continental populations from the 1,000 Genomes database phase 3. Linkage disequilibrium (LD) analysis of risk alleles was performed on all populations. Interleukin-1 receptor associated kinase 1 (*IRAK1*) and methyl CpG binding protein 2 (*MECP2*) mRNA levels were determined using real-time PCR.

**Results:**

Case-control analysis revealed genetic association with childhood-onset SLE for all 15 SNVs (OR = 1.49–1.75; *p* = 0.0095 to 1.81 × 10^–4^) and for the Xq28 risk haplotype (OR = 1.97, *p* = 4 × 10^–6^). Comparing with individuals of European ancestry (0.14–0.16), the frequencies of the risk alleles were significantly higher in the MEZ individuals (0.55–0.68) and even higher in Indigenous individuals (0.57–0.83). LD analysis indicated a differential haplotype structure within the Indigenous groups, which was inherited to the MEZ population as a result of genetic admixture. Individuals homozygous for the Xq28 risk haplotype exhibited decreased levels of both *MECP2A* and *B* transcripts.

**Conclusion:**

We found that the H1 risk haplotype differs in its conformation in the Mexican population. This difference could be attributed to positive selection within the Indigenous population, with its inheritance now having an autoimmune health impact in both the Mexican Indigenous and MEZ populations.

## Introduction

Systemic lupus erythematosus (SLE) (OMIM #152700) is an autoimmune disease that involves chronic multi-systemic inflammation, and increased production of type I interferon (IFN-I) in circulating mononuclear cells and peripheral tissues (1, 2). Approximately 20% of SLE cases are childhood-onset SLE, and these present a more severe illness and higher mortality rates than adult-onset SLE (3).

Systemic lupus erythematosus prevalence and severity vary among different ethnic groups, with greater frequencies observed in African and Hispanic populations (4, 5). Genome-wide association studies (GWAS) have identified over 100 SLE-associated loci. While many genetic variants are shared among different ethnicities, some appear to be more common in certain populations. This observation is strengthened by the finding of ancestry-specific genetic variants associated with SLE (6). Populations of Amerindian origin, such as the Mexican population, seem to have been under positive selection for several variants within genes related to IFN signaling pathways, as well as others involved in the immune response (7). For example, the risk haplotype TCA (rs2004640, rs2070197, and rs10954213) located in *IRF5*, which confers the highest reported risk for childhood-onset SLE, exhibits the highest frequency in the Mexican population (8).

It has been reported that SLE is independently associated with genes in the Xq28 region including the interleukin-1 receptor associated kinase 1 gene (*IRAK1*), and its adjacent methyl CpG binding protein 2 (*MECP2*) and transmembrane protein 187 (*TMEM187*) genes (9–11). *IRAK1* is a key regulator of the immune response, and is involved in the signaling pathways of the Toll-like receptors (TLRs) and IL-1 receptor (12–14). *MECP2* acts as a transcriptional modulator of methylation-sensitive genes, and interacts with the DNA methyltransferase DNMT1 for maintenance of DNA methylation (15). *TMEM187* encodes a multi-pass membrane protein, however, its biological function has not been yet determined (16). Single-nucleotide variants (SNVs) in these genes are reported to be in strong linkage disequilibrium (LD). Among the haplotypes in the Xq28 region, only the H1 haplotype containing the risk alleles exhibits a persistent association with SLE risk in all studied populations. Notably, the *TMEM187-IRAK1-MECP2* locus also confers a differential susceptibility for other autoimmune diseases, e.g., rheumatoid arthritis (RA) (16). These studies have shed light on the differences among SLE risk variants observed in patients with different ancestral backgrounds (6, 17).

In the present study, we aimed to investigate the association of 15 SNVs within the Xq28 risk locus with childhood-onset SLE, and to determine whether the risk haplotype is enriched in the Mexican Mestizo (MEZ) and Indigenous populations. We also evaluated the functional effect of the *IRAK1*-*MECP2* risk haplotype.

## Materials and methods

### Population cohorts

A cohort of 376 Mexican MEZ patients with childhood-onset SLE was recruited from two tertiary level institutions in Mexico City. From this cohort, a set of 225 trio families was used for transmission disequilibrium test (TDT) analysis. All patients were younger than 16 years old, and met the American College of Rheumatology (ACR) SLE classification criteria (18). The control group included 400 healthy sex-matched adults from Mexico City (MEZ). To compare the allele frequencies of the 15 SNVs from the Xq28 region of both the MEZ and Amerindian (AM) populations with those reported for other populations in the 1,000 Genomes database (1KGP) phase 3, we used imputed whole-exome sequencing from 1,074 AM individuals from the Metabolic Analysis in an Indigenous Sample (MAIS) cohort (median Amerindian ancestry of 0.96) (19, 20). To validate the imputed risk alleles, we genotyped tag SNVs in 300 AM individuals belonging to four of the most representative Mexican ethnic groups: Tarahumara from the North (N), Nahuatl from the Central East (CE), Zapoteco from the South (S), and Mayan from the Southeast (SE).

This study was conducted in accordance with the Declaration of Helsinki. All individuals signed an informed consent, and the study was approved by local ethics and research committees from all participating institutions. Parents provided consent for child participation, and all children older than 8 years old assented.

### Genotyping analysis

Genomic DNA was isolated from whole blood samples using the Gentra-Puregene kit (Qiagen, Valencia, CA, United States). We genotyped 15 SNVs using TaqMan Assays on Demand (Applied Biosystems, Foster City, CA, United States). The genotyped SNVs were rs2266890 and rs13397 located in *TMEM187*; rs3027898, rs2239673, rs763737, rs5945174, rs7061789, and rs1059702 located in *IRAK1*; and rs2075596, rs3027933, rs17435, rs1734787, rs1734791, rs1734792, and rs2239464 located in *MECP2*. The call rate was >98% for all SNVs. Genotypes were confirmed by randomly sequencing 10% of the samples using an automated ABI PRISM 310 Genetic Analyzer (Applied Biosystems, Life Technologies, CA, United States), which showed 100% reproducibility. To estimate ancestry in 320 SLE patients and 400 controls, we used the 6.0 SNV array (Affymetrix, Santa Clara, CA, United States) or GoldenGate genotyping assay (Illumina, San Diego, CA, United States), which contained 96 ancestry-informative markers (AIMs) validated in previous studies (21). The median Amerindian ancestry was 0.72 for cases and 0.66 for controls.

### Frequency analyses, case-control study, and haplotype analysis

The allele frequencies were compared between Mexican populations and other continental populations taken from the 1KGP phase 3, using a chi-square test in R v.3.1 (http://www.r-project.org). To validate the frequencies of the SNVs in Indigenous individuals, we selected rs1059702, rs2075596, rs2239464, rs2266890, and rs3027898, which we predicted to be tag SNVs. For the SLE case-control association, logistic regression was performed. The statistical power was computed using the Genetic Power Calculator program (22), assuming a disease prevalence of 0.06% (23) and a significance level of 0.05. The association was evaluated under an additive model, and was adjusted by sex and ancestry, using the first two principal components. A *p* value of <0.05 after Bonferroni multiple testing correction was considered statistically significant. All analyses were performed using PLINK v.1.9 software (24). Haploview v.4.2 program was used for LD calculation through pairwise *r*^2^ values, allele, and haplotype frequencies, and to construct haplotypes (25). LD plots were constructed for the Mexican Indigenous and MEZ populations, and imputation was performed using IMPUTE 2 (26), using the 1KGP phase 3 as a reference panel. Variants with an information score of >0.9 were included in the analysis.

### Real-time quantitative PCR (qPCR)

Total RNA was obtained from PMBCs collected from eight adult healthy control carriers of the Xq28 risk haplotype (H1) and 12 non-carriers, using Trizol reagent (Invitrogen, Carlsbad, CA, United States) according to the manufacturer’s instructions. RNA integrity was assayed by agarose gel electrophoresis. For the mRNA reverse transcription, 500 ng of total RNA, and oligo dT_18_ primer were used, along with the RevertAid enzyme (Thermo Fisher Scientific, Massachusetts, United States). We measured *IRAK1* expression using the probe Hs01018347_m1, and we measured the two *MECP2* isoforms (A and B) using the probes Hs00172845_m1 and Hs01598237_m1 (Thermo Fisher Scientific, Massachusetts, United States), respectively. The data were normalized using the *GAPDH* Endogenous Control TaqMan probe (Thermo Fisher Scientific, Massachusetts, United States). All qPCR assays were performed using a ViiA7™ Real Time system thermocycler (Thermo Fisher Scientific, Massachusetts, United States). Relative mRNA expression levels were calculated using the 2^–ΔCt^ method (27). Statistical analysis was performed using Student’s *t*-test, with GraphPad Prism software v.9.0 (GraphPad, San Diego, CA, United States). A *p* value < 0.05 was considered to indicate significant differences.

## Results

### Association of the Xq28 region with childhood-onset SLE

Based on the sample size, a statistical power of 80% was reached. The case-control analysis for each of the 15 analyzed single variants in the Xq28 region revealed that all of the tested alleles were significantly associated with childhood-onset SLE, with ORs ranging from 1.49 to 1.75 after correction for ancestry and sex (Table 1). We subsequently replicated this association using TDT analysis, by genotyping the 15 SNVs in 225 trios. We found that all risk alleles were over-transmitted, although only two SNVs located on *IRAK1* (rs763737 and rs59455174) and three located on *MECP2* (rs2075596, rs3027933, and rs17435) were significantly associated with childhood-onset SLE (Table 2). Furthermore, only the haplotype H1 was associated with susceptibility to childhood-onset SLE (OR = 1.97, *p* = 4 × 10^–6^) in the Mexican population (Table 3).

**TABLE 1:** Case-control allelic association.

SNV	Gene	Tested allele	Allele frequency		OR* (CI 95%)	*P* *
			**Controls**	**Cases**		
rs2266890	*TMEM187*	*T*	0.68	0.71	1.49 (1.10–2.03)	0.00950
rs13397	*TMEM187*	*A*	0.57	0.66	1.58 (1.18–2.13)	0.00251
rs3027898	*TMEM187/IRAK1*	*C*	0.61	0.71	1.74 (1.30–2.34)	0.00018
rs2239673	*IRAK1*	*C*	0.61	0.70	1.70 (1.26–2.33)	0.00066
rs763737	*IRAK1*	*G*	0.61	0.70	1.60 (1.18–2.17)	0.00228
rs5945174	*IRAK1*	*G*	0.60	0.69	1.66 (1.23–2.25)	0.00097
rs7061789	*IRAK1*	*G*	0.62	0.69	1.54 (1.14–2.09)	0.00488
rs1059702	*IRAK1*	*A*	0.56	0.66	1.63 (1.22–2.20)	0.00101
rs2075596	*MECP2*	*A*	0.55	0.65	1.57 (1.17–2.12)	0.00280
rs3027933	*MECP2*	*G*	0.57	0.64	1.56 (1.17–2.10)	0.00252
rs17435	*MECP2*	*T*	0.61	0.69	1.75 (1.31–2.36)	0.00018
rs1734787	*MECP2*	*C*	0.56	0.65	1.68 (1.26–2.24)	0.00038
rs1734791	*MECP2*	*T*	0.56	0.65	1.64 (1.24–2.19)	0.00059
rs1734792	*MECP2*	*A*	0.57	0.65	1.64 (1.22–2.20)	0.00092
rs2239464	*MECP2*	*A*	0.60	0.69	1.65 (1.22–2.24)	0.00123

Association of childhood-onset systemic lupus erythematosus (SLE) with the 15 single-nucleotide variants (SNVs) located in the Xq28 risk haplotype among Mexican patients. *The data were corrected for ancestry and sex.

**TABLE 2:** Transmission disequilibrium test (TDT) of the interleukin-1 receptor associated kinase 1 (*IRAK1*)/methyl CpG binding protein 2 (*MECP2*) single-nucleotide variant (SNV) alleles in Mexican systemic lupus erythematosus (SLE) families using haploview.

SNV	Allele	*T*	*U*	OR	*p*
rs2266890	*T*	41	35	1.17 (0.74–1.83)	0.491
rs13397	*A*	30	21	1.42 (0.81–2.49)	0.208
rs3027898	*C*	34	23	1.47 (0.87–2.50)	0.145
rs2239673	*G*	48	32	1.5 (0.95–2.34)	0.074
rs763737	*G*	48	26	1.84 (1.14–2.97)	0.011
rs5945174	*G*	44	27	1.62 (1.00–2.63)	0.044
rs7061789	*G*	48	32	1.5 (0.95–2.34)	0.074
rs1059702	*A*	38	26	1.46 (0.88–2.40)	0.134
rs2075596	*A*	54	35	1.54 (1.00–2.36)	0.044
rs3027933	*G*	54	35	1.54 (1.00–2.36)	0.044
rs17435	*A*	50	32	1.56 (1.00–2.43)	0.047
rs1734787	*C*	49	33	1.48 (0.95–2.30)	0.077
rs1734791	*A*	53	35	1.51 (0.98–2.32)	0.055
rs1734792	*C*	43	35	1.22 (0.78–1.91)	0.365
rs2239464	*A*	47	31	1.51 (0.96–2.38)	0.070

The frequency, chi-square, and *p* values are depicted for each haplotype.

**TABLE 3:** Haplotype frequencies in a case-control analysis.

Haplotype	Frequency		OR (CI 95%)	*p*
	**Controls**	**Cases**		
TACCGGGAAGTCTAA (H1)	0.47	0.61	1.97 (1.48–2.63)	4.00E-06
CGATAAACGCAAACG (H2)	0.24	0.22	0.79 (0.57–1.10)	0.160
TGATAAACGCAAACG (H3)	0.07	0.05	0.65 (0.33–1.28)	0.213
TAACGGGAAGTCTAA (H4)	0.05	0.02	0.09 (0.01–0.65)	0.017
CGCTAAACGCAAACG (H5)	0.04	0.02	0.58 (0.25–1.35)	0.209
TGCCGGGCGCTAACA (H6)	0.03	0.02	1.07 (0.47–2.45)	0.873

The frequency, chi-square, and *p*-values are depicted for each haplotype.

### Frequencies of variants in the Xq28 region within the Mexican population

We compared the allele frequencies of these SNVs among our 400 MEZ controls with the frequencies reported for other populations in the 1KGP phase 3. Only the South and East Asian populations exhibited greater allele frequencies than the MEZ population from our present study. The frequencies of all the analyzed risk alleles within our sample were higher compared with those reported for persons of Mexican Ancestry from Los Angeles, California, USA (MXL); were four times greater than in the Iberian Population in Spain (IBS), and were even greater than those reported in the African population (AFR) (Table 4 and Supplementary Table 1). To confirm whether these risk alleles were inherited by the MEZ from the Indigenous population, we used imputed whole-exome sequencing from Indigenous populations belonging to the MAIS Cohort (median Amerindian ancestry of 0.96). Genotype validation was performed using tag SNVs (Supplementary Table 2) in AM individuals belonging to four of the most representative Mexican ethnic groups: Tarahumara, Nahuatl, Zapoteco, and Mayan (which show a concordance of >99%). We found that the allele frequencies of the risk variants were all significantly enriched in the AM population, being some of the highest frequencies worldwide. Our analysis also revealed an increasing gradient from the north to the south of the Mexican territory (Supplementary Table 3).

**TABLE 4:** Minor allele frequencies from 15 single nucleotide variants (SNVs) within the Xq28 region in different populations.

Population	*TMEM187*		*IRAK1*						*MECP2*						
	**rs2266890**	**rs13397**	**rs3027898**	**rs2239673**	**rs763737**	**rs5945174**	**rs7061789**	**rs1059702**	**rs2075596**	**rs3027933**	**rs17435**	**rs1734787**	**rs1734791**	**rs1734792**	**rs2239464**
	***T***	***A***	***C***	***C***	***G***	***G***	***G***	***A***	***A***	***G***	***T***	***C***	***T***	***A***	***A***
MEZ*	0.68	0.57	0.61	0.61	0.61	0.60	0.62	0.56	0.55	0.57	0.61	0.56	0.56	0.57	0.60
AM*	0.79	0.66	0.73	0.74	0.73	0.73	0.73	0.68	0.67	0.67	0.72	0.69	0.69	0.69	0.72
MXL	0.53	0.39	0.45	0.45	0.45	0.45	0.45	0.40	0.39	0.40	0.47	0.41	0.42	0.42	0.47
AMR	0.48	0.42	0.48	0.49	0.49	0.49	0.49	0.43	0.41	0.42	0.51	0.42	0.44	0.44	0.50
EAS	0.73	0.70	0.80	0.80	0.80	0.80	0.80	0.78	0.78	0.78	0.79	0.79	0.79	0.77	0.80
SAS	0.61	0.58	0.65	0.65	0.65	0.65	0.65	0.61	0.61	0.61	0.61	0.64	0.61	0.60	0.64
IBS	0.16	0.14	0.15	0.15	0.15	0.15	0.15	0.14	0.14	0.16	0.16	0.15	0.16	0.16	0.16
EUR	0.19	0.15	0.18	0.18	0.18	0.18	0.18	0.15	0.14	0.15	0.19	0.15	0.16	0.15	0.19
AFR	0.09	0.03	0.47	0.47	0.47	0.47	0.48	0.03	0.04	0.15	0.64	0.08	0.26	0.22	0.41

*Present study.

### Haplotype structure of the Xq28 region in the Mexican population

**FIGURE 1:**
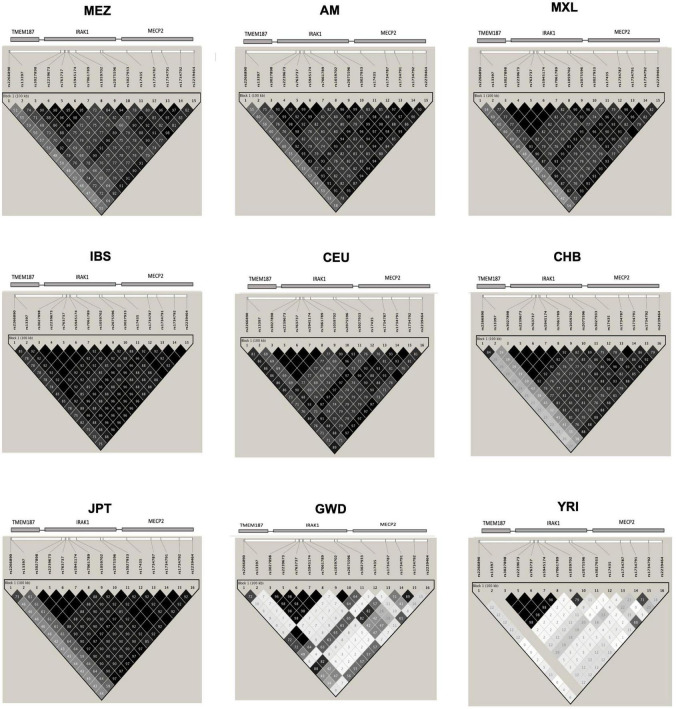
Linkage disequilibrium (LD) plot of the single-nucleotide variants (SNVs) in the Xq28 region. MEZ, Mestizo from the present study; AM, Amerindians from the present study; MXL, Mexican Ancestry from Los Angeles USA from the 1KGP phase 3; IBS, Iberian population in Spain from the 1KGP phase 3; CEU, Utah Residents with Northern and Western European ancestry from the 1KGP phase 3; CHB, Han Chinese in Beijing, China from the 1KGP phase 3; JPT, Japanese in Tokyo, Japan from the 1KGP phase 3; GWD, Gambian in Western Division, The Gambia-Mandinka from the 1KGP phase 3; YRI, Yoruba in Ibadan, Nigeria from the 1KGP phase 3. The color scale (black = significance; white = no significance) highlights the pair-wise D’, and the numbers on the boxes represent the pair-wise *r*^2^ between the SNVs that are marked on the top of the LD plot. The location of each SNV is indicated at the top of the LD plot.

Evaluation of the haplotype H1 structure in the MEZ and AM populations revealed that the SNVs located in *IRAK1–MECP2* were in strong LD, while those located in *TMEM187* exhibited lower LD values. Similar results were found in all Latin American populations and in East Asians (EAS) from the 1KGP phase 3. In contrast, in the IBS and CEU populations, the risk haplotype maintained a strong LD, including the variants in *TMEM187* (*r*^2^: IBS = 85 and 92; CEU = 81 and 69). Interestingly, African populations (YRI and GWD) did not show LD among most of the risk alleles in the Xq28 region, except in five (rs3027898, rs2239673, rs763737, rs5945174, and rs7061789) of the seven analyzed SNVs in *IRAK1*, which were observed to be in complete LD in all populations (Figure 1).

**FIGURE 2:**
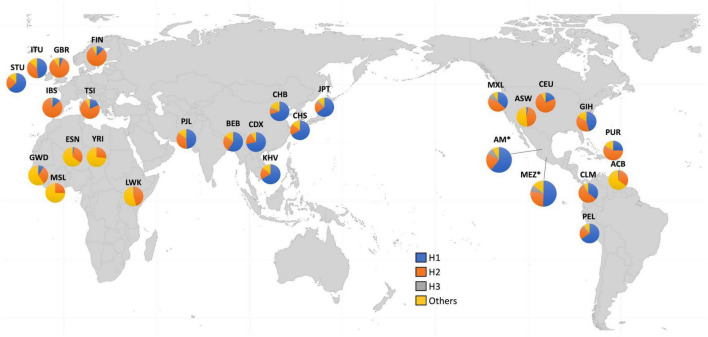
Worldwide frequencies of Xq28 haplotypes. Haplotype H1–H3 frequencies are represented for each of the included 1KGP phase 3 populations.

Because of these specific population differences in the structure of the Xq28 region, we were able to observe a variable number of haplotypes in this region with a frequency ≥ 0.01. For example, five haplotypes were observed in the MEZ and MXL populations, four in AM, three in IBS, and 14 in African populations. The risk haplotype H1 was found at a frequency of 0.61 in AM, 0.49 in MEZ, and 0.13 in IBS, while it was absent in African populations, such as the Mende in Sierra Leona (MSL) and Yoruba in Ibadan Nigeria (YRI) (Figure 2).

### Effect of the Xq28 risk haplotype on *MECP2* and *IRAK1* transcript levels

**FIGURE 3:**
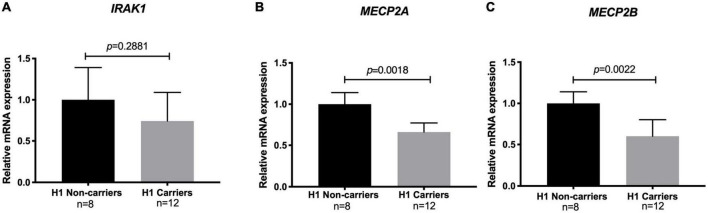
Relative mRNA levels of methyl CpG binding protein 2 (*MECP2*) isoforms and interleukin-1 receptor associated kinase 1 (*IRAK1*) among risk haplotype Xq28 carriers. **(A)** *IRAK1*. **(B)** *MECP2* isoform A. **(C)** *MECP2* isoform B. Transcript expression was normalized to GAPDH (log_2_ fold change > 2 and adjusted *p* < 0.05).

To examine the effect of the risk haplotype on the expression of *IRAK1* and the two *MECP2* isoforms in healthy control H1 haplotype carriers and non-carriers, we performed real-time quantitative PCR (qPCR). Notably, the levels of both the *MECP2A* and *B* transcript isoforms were significantly lower (40%) in the risk haplotype carriers. The analysis of *IRAK1* transcripts did not show significant differences between carriers and non-carriers (Figure 3).

## Discussion

Accumulating evidence indicates that the complex clinical outcomes and genetic factors associated with SLE vary depending on ancestral background of each population (4, 6, 28). These observations support the cumulative hit hypothesis for autoimmune diseases, which states that immune dysregulation will ultimately result in disease development when an individual with a high number of risk variants is exposed to sufficient environmental risk factors (28). Hence, studying the genetic particularities for each population can be helpful for better understanding autoimmune diseases. The Mexican MEZ genetic structure is substantially influenced by its Amerindian inheritance (29). For instance, the enrichment of certain risk loci in the Mexican Amerindian and MEZ populations, such as the *IRF5* TCA risk haplotype, could partly explain the high prevalence of autoimmune diseases in the Mexican population (8).

The Xq28 region harbors three genes that have been associated with SLE: *IRAK1* and its adjacent genes *TMEM187* and *MECP2*. To deepen our knowledge of the genetic contribution of the Xq28 region to childhood-onset SLE pathogenesis, here we genotyped 15 SNVs within this region, investigated their association with childhood-onset SLE, and examined the haplotype structure differences among various populations. We also evaluated the functional effect of the risk haplotype. We found that childhood-onset SLE was significantly associated with all of the tested alleles (OR = 1.49–1.75), and with the H1 haplotype spanning *TMEM187, IRAK1*, and *MECP2* (OR = 1.97) (Tables 1, 3). This finding is in agreement with previously reported associations of these variants with SLE among individuals from different ancestral groups (30).

Ethnicity-based genetic particularities were found for all analyzed risk alleles, exhibiting the highest frequencies in EAS, followed by all indigenous groups, SAS, and MEZ (Table 4 and Supplementary Table 3). Notably, the frequency of the risk H1 haplotype in the MEZ population (0.47) was closer to that in the AM population (0.65) than to that in the IBS population (0.13) (Tables 3, 4 and Supplementary Tables 1, 3). This finding is in contrast to the relationship observed for alleles harbored on autosomes, which in MEZ usually exhibit intermediate frequencies between AM and IBS (31–34). We also found that the variants located in *TMEM187* (rs2266890 and rs13397) exhibited a significantly decreased LD *r*^2^ value in both the MEZ and AM populations, while the LD *r*^2^ value remained high for all analyzed SNVs in the IBS population (Figure 1). A similar trend was also observed in other Latin American populations like Peruvians, who also show a high degree of Native American ancestry (Figure 2). This is in line with previous observations that MEZ exhibit elevated levels of Amerindian ancestry on chromosome X compared to autosomes (35). Altogether, our results suggest that the main contribution to H1 haplotype frequency and structure in the MEZ population derives from their Amerindian ancestral origin, as a result of the admixture patterns observed in the colonial period more than 500 ybp, which mainly involved Indigenous females and European males (36, 37).

As previously reported (30, 38), we observed an effect of the H1 risk haplotype only on both *MECP2A* and *MECP2B* transcripts, with significantly lower levels in H1 carriers (Figure 3). This is relevant due to the role of MECP2 on DNA methylation, which could be related to the DNA hypomethylation described in SLE patients, especially in T-cells (39–41). Although this effect was not observed for *IRAK1* mRNA levels, Kaufman et al. previously reported that the causal variant rs1059792, located in exon 5 of *IRAK1*, results in an amino acid change (S196F) that impairs the interaction between IRAK1 and NFκB, leading to immune response dysregulation by increased NFκB activity and an enhanced inflammatory response (30, 42).

In summary, here we report that the Xq28 risk haplotype confers susceptibility to childhood-onset SLE in the Mexican population. The risk haplotype exhibited decreased LD values for two of the studied SNVs, both located in *TMEM187*, among the MEZ and Indigenous populations, but not in populations without Amerindian ancestry from the 1KGP phase 3. The Xq28 risk haplotype structure, and its high allele frequency observed in MEZs and in all Indigenous groups, suggest an Amerindian origin. These could be the result from both the selection of variants in immune-related genes, and the high Native American ancestry displayed on the X-chromosome in the MEZ population. These observations could be due to the demographic events that occurred during the European colonization, and could explain the increased prevalence of autoimmune diseases, such as SLE, in Hispanic populations.

## Data availability statement

The original contributions presented in the study are publicly available. This data can be found at: Flannick et al. (43).

## Ethics statement

The studies involving human participants were reviewed and approved by the Instituto Nacional de Medicina Genómica Ethics and Research committees. Written informed consent to participate in this study was provided by the participants or their legal guardian/next of kin.

## Author contributions

CC-C and LO: study conception and design. VB: sample and clinical information collection. HG-O, FB-O, MF-H, MM-R, and CC-C: experiments and data analyses. HG-O, FB-O, AM-H, VB, CC-C, and LO: interpretation of data. HG-O, FB-O, CC-C, and LO: writing manuscript. MF-H, MM-R, AM-H, and VB: critical revision of the manuscript. HG-O, CC-C, and LO: obtaining funding and supervision. All authors contributed to the article and approved the submitted version.
