# Supplementary material for: Ancestry-dependent genetic structure of the Xq28 risk haplotype in the Mexican population and its association with childhood-onset systemic lupus erythematosus
Source: Front Med (Lausanne). 2023 Jan 12;9:1044856. doi: 10.3389/fmed.2022.1044856 (PMC9877425; doi:10.3389/fmed.2022.1044856)
Supplement: Supplementary file 1 [file Table_1.docx]

**Supplementary Material**

**Supplementary Table 1.** Allele frequency comparison between Mestizo healthy controls and Continental Populations (*X*^2^, p-value).

**Supplementary Table 2.** Tag SNVs.

**Supplementary Table 3.** Minor allele frequencies from rs2266890, rs3027898, rs1059702, rs2075596 and rs2239464 SNVs within the Xq28 region in Indigenous groups.

**N: North, CE: Central East, S: South, SE: South East**
